# Macronutrient intake and frailty: the Rotterdam Study

**DOI:** 10.1007/s00394-019-02131-0

**Published:** 2019-11-14

**Authors:** Eline Verspoor, Trudy Voortman, Frank J. A. van Rooij, Fernando Rivadeneira, Oscar H. Franco, Jessica C. Kiefte-de Jong, Josje D. Schoufour

**Affiliations:** 1 Department of Epidemiology, Erasmus University Medical Center, Rotterdam, The Netherlands; 2 Department of Public Health, Erasmus University Medical Center, Rotterdam, The Netherlands; 3 Department of Internal Medicine, Erasmus University Medical Center, Rotterdam, The Netherlands; 4 Department of Public Health and Primary Care, Leiden University Medical Center/LUMC Campus, The Hague, The Netherlands; 5 Institute of Social and Preventive Medicine (ISPM), University of Bern, Bern, Switzerland; 6 Faculty of Sports and Nutrition, ACHIEVE-Centre of Applied Research, Faculty of Health, Amsterdam University of Applied Sciences, Amsterdam, The Netherlands

**Keywords:** Older adults, Frailty, Frailty index, Macronutrient intake

## Abstract

**Purpose:**

To investigate the longitudinal association between the macronutrient composition of the diet and frailty.

**Methods:**

Data were obtained from 5205 Dutch middle-aged and older adults participating in the Rotterdam Study. Frailty was measured using a frailty index based on the accumulation of 38 health-related deficits, score between 0 and 100, and a higher score indicating more frailty. Frailty was assessed at baseline and 11 years later (range of 23 years). Macronutrient intake was assessed using food-frequency questionnaires. The association between macronutrients and frailty over time was evaluated using multivariable linear regression, adjusted for the frailty index at baseline, energy intake, and other relevant confounders. All analyses were performed in strata of BMI.

**Results:**

Median frailty index score was 13.8 points (IQR 9.6; 19.1) at baseline and increased by a median of 2.3 points (IQR − 2.0; 7.6) after 11 years. Overall, we found no significant associations between intake of carbohydrates or fat and frailty over time. We did observe a significant positive association between an iso-energetic intake of 10 g protein and frailty over time (*β* 0.31 (95% CI 0.06; 0.55)) which was mainly driven by animal protein (*β* 0.31 (95% CI 0.07; 0.56)). It did not depend on whether it was substituted fat or carbohydrates.

**Conclusions:**

Our findings suggest that a reduction in the intake of animal protein may improve the overall health status over time in a relatively healthy population. More research is needed on the optimal macronutrient composition of the diet and frailty in more vulnerable populations.

**Electronic supplementary material:**

The online version of this article (10.1007/s00394-019-02131-0) contains supplementary material, which is available to authorized users.

## Introduction

The rapid aging of our population is a major public health issue [1]. A longer lifespan is often accompanied by an increased risk of disability and mortality, including the appearance of chronic diseases such as cardiovascular disorders, cancer, stroke, and dementia [2]. In addition to the focus on chronic diseases, a high amount of research tries to capture overall health. Overall health is determined by the accumulation of a wide range of health problems, including symptoms, signs, diseases, and disabilities [3], and not merely the absence of chronic diseases [4].

One way to assess overall health is via frailty, generally described as a non-specific state of homeostatic dysregulation in multiple systems, and vulnerability to stressors, such as illness, injury, or psychological stress [5, 6]. Frailty is a strong predictor for adverse events, including disability, institutionalization, hospitalization, and mortality [7, 8]. There are two well-known operationalizations of frailty: physical frailty and multidimensional frailty. Physical frailty, based on the presence of at least three of the following five criteria: weight loss, weak grip strength, exhaustion, slow gait speed, and low physical activity, mainly focused on predefined physical variables [5]. While multidimensional frailty covers a broad range of health domains, combining indicators on cognition, disabilities, biochemical abnormalities, and diseases [3, 9]. Indicators on separate health domains have only small effects on health; their cumulative effect becomes significant [10].

## Multidimensional frailty focuses on a more holistic approach to treatment, rather single health deficits

For healthy aging, it is important to counteract the onset and progression of frailty. Different lifestyle factors play an important role in the prevention of frailty. One important modifiable factor is nutrition, by providing energy which is important for the overall homeostasis and by proving essential nutrients, necessary for the maintenance of bodily and organ functions [11]. So far, a recent literature review showed that most studies have focused on the association between protein and physical frailty [12]. High protein intake is shown to be beneficiary for physical frailty including muscle mass and muscle strength [13]. Nonetheless, far too little attention has been put to the association between macronutrients in general and more holistic approaches such as the frailty index. Considering multidimensional frailty, macronutrients intake might be beneficiary for some health domains but harmful for other health domains. For example, on one hand, a high protein diet is associated with higher satiety and lower total caloric intake, and lower body weight, and less body adiposity [14–17]. However, on the other hand, it is suggested that high protein intake might be harmful to kidney function [18]. Also, two systematic reviews concluded that high protein intake but a low carbohydrate intake was associated with higher all-cause mortality risk [19, 20]. Similar, a high carbohydrate or fat intake is associated with an increased coronary heart disease risk and a higher body mass index [21]; on the other hand, overweight might have a lower all-cause-mortality compared to normal weight at an older age [22].

To our knowledge, only a few studies investigated the association between diet and the frailty index, all focused on diet quality. These studies showed that better diet quality is associated with less frailty [23–25]. No studies are available on macronutrient intake and multidimensional frailty. We hypothesize that the macronutrient composition of the diet is of influence on the frailty index. The aim of the current study is to examine the longitudinal association of macronutrient intake with the frailty index, taking total energy intake and the overall macronutrient composition of the diet into account.

## Methodology

### Study design and participants

Data were obtained from the Rotterdam Study (RS), a population-based prospective cohort of middle-aged and older adults. The design of the Rotterdam Study has extensively been described elsewhere [26]. Briefly, the Rotterdam Study started in 1990, inviting all residents aged 55 years and over (*n *= 10,235) in a specific suburb of Rotterdam, from which 7983 took part in the RS’s first cohort (RS-I). The study was extended with new participants in 2000, inviting all residents aged 55 years and over or who moved into the study area (RS-II; *n* = 3011). In 2006, the study was extended with a third cohort, inviting all residents aged 45 years and over (RS-III; *n* = 3932). Data collection for all cohorts at baseline included questionnaires and an interview at home (2 h) by trained research assistants on among others activities of daily living, current health status, medical history, diet, medication use, smoking, highest obtained education, and physical activity. Additionally, participants visited our dedicated study center in the center of their district where physical examinations took place; stressing on body size, imaging, collection of body fluids, physical functioning, and cognitive performance. Examinations were repeated in each cohort every 3–5 years. For the current study, we excluded participants if their energy intake was implausible, having an estimated energy intake lower than 500 kcal or higher than 5000 kcal per day. Participants were included with a valid frailty index at baseline and follow-up, resulting in a total study population 5205 participants.

### Dietary assessment

Dietary intake was assessed using validated Food-Frequency Questionnaires (FFQ), described in detail elsewhere [27]. Briefly, in RS-I-1 and RS-II-1, participants completed a checklist at home about foods and drinks which they consumed at least twice a month during the preceding year. Thereafter, trained dietitians interviewed the participants at the research center, using a validated, computerized 170-item semi-quantitative FFQ. This FFQ was previously validated against fifteen 24 h food records and four 24 h urinary urea excretion samples in a subsample of the RS, and showed good validity for macronutrient intakes (*r* for protein 0.61, *r* for fat 0.70, and *r* for carbohydrates 0.72) [28]. In RS-III-1, dietary intake was measured with a self-administrated, semi-quantitative FFQ. This FFQ was validated, in two other Dutch populations using a 9-day dietary record and a 4-week dietary history, and showed moderate-to-good validity for macronutrient intakes (*r* for protein 0.61, *r* for fat 0.47, and *r* for carbohydrates 0.71) [29]. This FFQ included 389 items on the frequency and amount of consumed food items over the last month. For the calculation of macronutrient intakes, the Dutch food composition database (NEVO) was used [30]. We calculated intake of the following macronutrients which were included in the analyses: total carbohydrates, mono- and disaccharides, polysaccharides, total fat, saturated fatty acids mono-unsaturated fatty acids, poly-unsaturated fatty acids, total protein, animal protein, and vegetable protein. Additionally, we calculated the intake of dietary fibers and alcohol intake in energy percentages, which were included as confounders in the analyses. Participants were excluded from the analyses.

### Frailty index

Frailty was derived from the frailty index, previously designed for and validated in the Rotterdam Study [31]. The frailty index was assessed in RS-I-3, RS-I-5, RS-II-1, RS-II-2, RS-III-1, and RS-III-2 [26]. Of the original Rotterdam Frailty index (45 items), seven items (vitamin D, sex hormone binding globulin, mobility, uric acid, pro-brain natriuretic peptide, homocysteine, and C-reactive protein) were removed, because these items were not assessed at follow-up. De Haas et al. showed that the original Rotterdam Study frailty index and the adapted version of the frailty index (*r* = 0.98) had no major differences in frailty [25]. The frailty index consisted of 38 deficits, covering different health domains: functional status (*n* = 13), cognition (*n* = 6), diseases (*n* = 6), health conditions (*n* = 6), nutritional status (*n* = 3), and mood (*n* = 4). Deficits were dichotomized or categorized, based on previously predefined cut-off values [31] into a score ranging from 0 (deficit not present) till 1 (deficit present). Per person, the sum of all deficits was divided by the total number of deficits, resulting in a score ranging from 0 (no deficits present, least frail) till 1 (all deficits present, extremely frail). For the interpretation of the data, the frailty index score was multiplied by 100, resulting in a range from 0 to 100.

### Other study parameters

Smoking status was classified as never, former, or current smoker. Level of education was determined by the highest attained education and recorded in four categories: low (primary education and lower vocational education), middle (secondary general education and secondary vocational education), middle–high (higher general education), and high (higher vocational education or university education). Net monthly household income was classified as low (< 1200€), middle (1200–2100€), and high (≥ 2100€). For RS-I and RS-II, physical activity was measured with an adapted version of the Zutphen Physical Activity Questionnaire [32], whereas for RS-III, the validated LASA physical activity Questionnaire (LAPAQ) [33] was used. Metabolic equivalents of task (MET) scores were calculated for the physical activities, weighted by their intensity, according to the compendium of physical activities 2011 [34]. Subsequently, MET hours per week were calculated for each participant. To take the differences of the questionnaires into account, MET hours per week were standardized by cohort (RS-I, RS-II, and RS-III) by calculating *Z* scores. Body height and weight were measured standing in light clothes, without shoes. BMI was calculated as weight divided by height squared (kg/m^2^) and defined as: normal weight (BMI ≤ 25 kg/m^2^), overweight (BMI 25–30 kg/m^2^), and obese (BMI > 30 kg/m^2^).

### Statistical analysis

Baseline characteristics of the study population were provided as the median and interquartile range (IQR) and as frequency (percentage). Based on literature [22, 35] and a statistically significant interaction between carbohydrate, fat, and protein intake and BMI (*P* for interaction < 0.01), BMI was considered an effect modifier and all results are presented by the total population and by strata of BMI. Differences in baseline characteristics between strata of BMI were assessed by analysis of variance or Chi-square test.

The association between macronutrients and the frailty index was assessed using multivariable linear regression analyses. In all models, the frailty index at follow-up was included as the dependent variable and macronutrient intakes as the independent variables. Two methods to adjust for total energy intake were used. First, we applied the nutrient residual method and included the macronutrient intake adjusted for total energy intake, modeled as an increase of 10 g/day macronutrient [36]. Coefficients can be interpreted as the difference in frailty index score per increase of 10 g/day intake of a specific macronutrient keeping energy intake constant (iso-energetic) and as a result lower intake in one or more of the other macronutrients. Second, we applied the nutrient density method by including macronutrient separately (per five energy percentage) as well as summed to represent total energy intake. By excluding, for example, protein intake from the analysis, the beta for each macronutrient represented the change in frailty index for a 5E% higher intake of that particular macronutrient and a concomitant lower intake of protein.

Based on previous literature [24, 25], three models were built: a basic model (model 1) adjusted for total energy intake (continuous), age (continuous), sex (categorical), length of follow-up (continuous), frailty index at baseline (continuous), and cohort (categorical). A confounder model (model 2) was additionally adjusted for education (categorical), smoking status (categorical), physical activity (continuous), income (categorical), living situation (categorical), occupational situation (categorical), and fiber and alcohol intake (in energy percentages, continuous) [37–39]. Finally, an intermediate model (model 3) was created which was additionally adjusted for BMI (continuous), because we hypothesized that BMI could be both a confounder and/or mediator in these associations. Analyses with specific subcategories of macronutrients were additionally adjusted for the other subcategories in energy percentages (e.g., animal protein was adjusted for vegetable protein intake and vice versa).

We performed several sensitivity analyses to test the robustness of the results. First, we applied the energy decomposition method to take total energy intake into account [40]. More details on this method are described elsewhere [36]. Second, effect modification was explored for age and sex [41–43], by adding interaction terms (macronutrient × effect modifier). Third, we excluded all deficits from the frailty index related to nutritional components (BMI, high-density lipoprotein, and hyperlipidemia) to evaluate if these deficits explained a possible association between macronutrients and frailty.

To impute missing values on the covariates, we constructed a multiple imputation procedure (*n* = 10 imputation sets). Results were presented by pooled analyses from multiple imputation data [44] and presented as betas (*β*) and 95% confidence intervals (95% CI). Statistical analyses were executed using IBM’s SPSS Statistics Version 24. Statistical tests were two-tailed.

## Results

### Characteristics of the study population

Of all 5205 participants, 59% were women and the median age of the population was 60 years (IQR 56; 63) (Table 1). The median energy intake was 2077 kcal (IQR 1727; 2511) of which, respectively, 44, 34, and 16 energy percentage of carbohydrate, fat, and protein. The median frailty index score at baseline was 13.8 points (IQR 9.6; 19.1), and on average, the frailty index increased by 2.3 points (IQR − 2.0; 7.6) after on average of 10.6 years of follow-up (range of 23 years). The frailty index at follow-up for the normal weight, overweight, and obese group was, respectively, 14.0 (8.8; 20.9), 15.6 (10.9; 22.3), and 19.7 (14.0; 27.5).

**Table 1:** Baseline characteristics of 5205 Dutch middle-aged and older adults

Baseline characteristics	All	BMI			*p* value^†^
	Total population (*n* = 5205)	Normal weight (*n* = 1556)	Overweight (*n* = 2464)	Obese (*n* = 1185)	
Frailty index, score	13.8 (9.6–19.1)	11.4 (7.5–15.6)	13.6 (9.9–18.4)	18.0 (13.6–23.2)	< 0.01
Sex, *n* (%)					
Women	3085 (59%)	989 (37%)	1146 (46%)	407 (34%)	< 0.01
Men	2120 (41%)	567 (63%)	1318 (54%)	778 (66%)	
Age (years)	59.6 (56.3–62.8)	59.1 (55.8–62.5)	59.7 (56.5–62.9)	59.7 (56.6–62.9)	0.01
≤ 60	2817 (54%)	894 (57%)	1308 (53%)	615 (52%)	< 0.01
> 60	2388 (46%)	662 (43%)	1156 (47%)	570 (48%)	
Smoking, *n* (%)					
Never smoker	1676 (32%)	506 (33%)	780 (32%)	389 (33%)	< 0.01
Former smoker	2480 (48%)	687 (44%)	1211 (49%)	583 (49%)	
Current smoker	1049 (20%)	363 (23%)	473 (19%)	213 (18%)	
Occupational situation, *n* (%)					
Work or voluntary work	2352 (45%)	718 (46%)	1122 (45%)	512 (43%)	< 0.01
Unemployed	247 (5%)	74 (5%)	117 (5%)	56 (5%)	
Retired	1632 (31%)	477 (31%)	789 (32%)	366 (31%)	
Househusband or housewife	974 (19%)	287 (18%)	435 (18%)	251 (21%)	
Education, *n* (%)					
Primary education	488 (9%)	142 (9%)	205 (8%)	141 (12%)	< 0.01
Lower education	2126 (41%)	601 (39%)	1011 (41%)	514 (43%)	
Intermediate education	1525 (29%)	436 (28%)	743 (30%)	346 (29%)	
Higher education	1066 (21%)	377 (24%)	505 (21%)	184 (16%)	
Income					
Low (< 1200€/month)	1023 (20%)	291 (19%)	463 (19%)	270 (23%)	< 0.01
Middle (1200–2100€/month)	1886 (36%)	588 (38%)	871 (35%)	427 (36%)	
High (≥ 2100€/month)	2296 (44%)	677 (43%)	1130 (46%)	488 (41%)	
Living situation, *n* (%)					
Independent	4929 (95%)	1483 (95%)	2336 (95%)	1110 (94%)	< 0.01
Dependent	276 (5%)	73 (5%)	128 (5%)	75 (6%)	
Physical activity, METh/week	70 (40–103)	75 (46–106)	71 (41–104)	63 (32–96)	< 0.01
Energy intake (kcal/day)	2077 (1727–2511)	2112 (1758–2542)	2098 (1755–2529)	1977 (1630–2392)	< 0.01
Macronutrient intake					
Carbohydrates, E%	44 (39–48)	45 (40–49)	43 (39–48)	43 (38–48)	< 0.01
Mono- and disaccharides, E%	24 (19–32)	24 (19–30)	23 (19–32)	23 (18–33)	< 0.01
Polysaccharides, E%	22 (19–25)	22 (19–25)	22 (19–25)	22 (19–25)	0.18
Dietary fiber, E%	4 (4–5)	4 (4–6)	4 (4–5)	5 (4–5)	< 0.01
Fat, E%	34 (30–39)	34 (30–38)	34 (30–39)	34 (30–39)	< 0.01
Saturated fatty acids, E%	13 (11–15)	13 (11–15)	13 (11–15)	13 (11–15)	< 0.01
Mono-unsaturated fatty acids, E%	11 (10–13)	11 (10–13)	11 (10–13)	11 (10–13)	0.37
Poly-unsaturated fatty acids, E%	7 (6–8)	7 (6–9)	7 (6–8)	7 (5–8)	< 0.01
Protein, E%	16 (14–18)	16 (14–17)	16 (15–18)	17 (15–19)	< 0.01
Animal protein, E%	10 (8–12)	9 (8–11)	10 (8–12)	11 (9–13)	< 0.01
Vegetable protein, E%	6 (5–7)	6 (5–7)	6 (5–7)	6 (5–7)	< 0.01
Alcohol, E%	2 (0–6)	2 (0–6)	3 (0–7)	2 (0–6)	< 0.01

Data are presented as median (interquartile range) or as frequency (percentage)

*BMI* body mass index, *METh* metabolic equivalent of task in hours, *E%* energy percentage

^†^Analysis of variance for continuous variables and Chi-square test for categorical variables. Frailty index: an instrument based on the accumulation of health deficits including age- and health-related symptoms, signs, diseases, disabilities, and laboratory measurements

### Macronutrients and frailty

By applying the nutrient residual method, after adjustment for confounders, total carbohydrate intake was not associated with frailty over time (Table 2) and also mono- and polysaccharides (Table 3) were not associated. Total fat, saturated, and poly-unsaturated fatty acids were not associated with frailty over time, but mono-unsaturated fatty acids was associated with more frailty over time in the total population [*β* 0.45 (95% CI 0.10; 0.81)]. Protein was associated, which was mainly by animal protein, with higher frailty levels over time, but only in the normal weight group [*β* 0.31 (95% CI 0.07; 0.56)] and not in the overweight or obese groups. The mediation model including BMI did not alter the results. By applying the nutrient density method, the direction of the associations remained mainly similar. A significant association between higher protein intake at the expense of carbohydrates and more frailty over time was observed [*β* 3.44 (95% CI 0.69; 6.19)], only in the normal weight group and not in the overweight or obese groups (Table 4). Also, a significant association was observed between higher protein intake at the expense of fat and more frailty over time [*β* 3.09 (95% CI 0.14; 6.04)] in the normal weight group, not in higher BMI groups.

**Table 2:** Longitudinal association between macronutrient intake and the frailty index using nutrient residual method in a Dutch middle-aged and older population

Macronutrient	Population	Model 1		Model 2		Model 3	
		*β*	95% CI	*β*	95% CI	*β*	95% CI
Carbohydrates (per 10 g/day)	Total population	− 0.07*	**− 0.11; − 0.02**	− 0.05	− 0.10; 0.003	− 0.03	− 0.09; 0.02
	Normal weight	− 0.11*	**− 0.19; − 0.02**	− 0.07	− 0.17; 0.03	− 0.07	− 0.16; 0.03
	Overweight	− 0.06	− 0.13; 0.01	− 0.02	− 0.10; 0.07	− 0.01	− 0.09; 0.07
	Obesity	− 0.02	− 0.11; 0.07	− 0.06	− 0.16; 0.04	− 0.05	− 0.15; 0.06
Fat (per 10 g/day)	Total population	0.15	− 0.04; 0.24	0.11	− 0.13; 0.23	0.09	− 0.28; 0.22
	Normal weight	0.16	− 0.05; 0.37	0.06	− 0.17; 0.28	0.06	− 0.17; 0.28
	Overweight	0.17	− 0.01; 0.34	0.11	− 0.08; 0.30	0.10	− 0.10; 0.29
	Obesity	0.11	− 0.10; 0.32	0.17	− 0.06; 0.40	0.16	− 0.08; 0.39
Protein (per 10 g/day)	Total population	− 0.001	− 0.13; 0.13	0.07	− 0.06; 0.20	0.01	− 0.13; 0.14
	Normal weight	0.22	− 0.03; 0.46	0.31*	**0.06; 0.55**	0.30*	**0.05; 0.55**
	Overweight	− 0.12	− 0.32; 0.07	− 0.07	− 0.26; 0.13	− 0.09	− 0.28; 0.11
	Obesity	− 0.14	− 0.40; 0.12	− 0.11	− 0.37; 0.16	− 0.16	− 0.43; 0.11

Values represent the difference in frailty index score per every increase of 10 g macronutrient intake, keeping the energy intake constant (iso-energetic) with their corresponding 95% Confidence Intervals (CI). Model 1 (basic model) was adjusted for age (continuous), sex (categorical), length of follow-up (continuous), frailty index at baseline (continuous), cohort (categorical), and kcal (continuous). Model 2 (confounder model) was additionally adjusted for education (categorical), physical activity (continuous), income (categorical), living situation (categorical), occupational situation (categorical), fiber intake (continuous), and alcohol intake (continuous). Model 3 (intermediate model) was additionally adjusted for BMI (continuous)

*Statistically significant at a *p* value < 0.05

**Table 3:** Longitudinal association between macronutrient intake and the frailty index using nutrient residual method in a Dutch middle-aged and older population divided into macronutrient subcategories

Type of macronutrient	Type of sub-macronutrient	All		BMI					
		Total population (*n* = 5205)		Normal weight (*n* = 1558)		Overweight (*n* = 2462)		Obese (*n* = 1185)	
		*β*	95% CI	*β*	95% CI	*β*	95% CI	*β*	95% CI
Carbohydrate (per 10 g/day)	Mono- and disaccharides	− 0.01	− 0.04; 0.03	-0.01	− 0.07; 0.06	0.004	− 0.05; 0.06	− 0.03	− 0.10; 0.04
	Polysaccharides	− 0.04	− 0.11; 0.04	− 0.11	− 0.25; 0.03	0.01	− 0.11; 0.12	− 0.001	− 0.16; 0.16
Fat (per 10 g/day)	Saturated fatty acids	− 0.09	− 0.39; 0.22	0.20	− 0.35; 0.75	− 0.19	− 0.67; 0.30	− 0.29	− 0.89; 0.30
	Mono-unsaturated fatty acids	0.45*	**0.10; 0.81**	0.09	− 0.53; 0.69	0.48	− 0.05; 1.02	0.72	− 0.04; 1.47
	Poly-unsaturated fatty acids	− 0.15	− 0.50; 0.21	− 0.20	− 0.80; 0.38	− 0.09	− 0.61; 0.45	0.08	− 0.76; 0.93
Protein (per 10 g/day)	Vegetable protein	0.03	− 0.32; 0.38	0.06	− 0.55; 0.66	0.07	− 0.47; 0.60	− 0.05	− 0.80; 0.70
	Animal protein	0.07	− 0.06; 0.20	0.31*	**0.07; 0.56**	− 0.07	− 0.26; 0.13	− 0.11	− 0.37; 0.16

Values represent the difference in frailty index score per every increase of 10 g macronutrient intake, keeping the energy intake constant (iso-energetic) with their corresponding 95% confidence intervals (CI). All models were adjusted for age (continuous), sex (categorical), length of follow-up (continuous), frailty index at baseline (continuous), cohort (categorical), kcal (continuous), education (categorical), physical activity (continuous), income (categorical), living situation (categorical), occupational situation (categorical), fiber intake (continuous), and alcohol intake (continuous)

*Statistically significant at a *p* value < 0.05

**Table 4:** Longitudinal association between macronutrient intake and the frailty index using nutrient density method in a Dutch middle-aged and older population

		All		BMI					
Type of macronutrient		Total population (*n* = 5205)		Normal weight (*n* = 1559)		Overweight (*n* = 2463)		Obese (*n* = 1183)	
Nutrient substitution		*β*	95% CI	*β*	95% CI	*β*	95% CI	*β*	95% CI
↑ Fat	↓ Carbohydrate^1^	0.37	− 0.32; 1.06	0.35	− 0.89; 1.59	0.14	− 0.93; 1.21	0.70	− 0.71; 2.10
↑ Protein	↓ Carbohydrate^2^	0.84	− 0.60; 2.28	3.44*	**0.69; 6.19**	− 1.15	− 3.38; 1.09	− 0.40	− 3.20; 2.50
↑ Protein	↓ Fat^3^	0.47	− 1.07; 2.01	3.09*	**0.14; 6.04**	− 1.29	− 3.66; 1.08	− 1.10	− 4.16; 1.95

Values represent the difference in frailty index score per every increase of 5 E% macronutrient intake, and a concomitant lower intake of the substitution macronutrient, with their corresponding 95% Confidence Intervals (CI). All models were adjusted for age (continuous), sex (categorical), length of follow-up (continuous), frailty index at baseline (continuous), cohort (categorical), kcal (continuous), education (categorical), physical activity (continuous), income (categorical), living situation (categorical), occupational situation (categorical), fiber intake (continuous), and alcohol intake (continuous)

*Statistically significant at a *p* value < 0.05

### Sensitivity analyses

First, we applied the energy decomposition method to take total energy intake into account (S1). In line with our main analyses, no associations were observed or carbohydrates or fat, and higher intake of protein was associated with more frailty over time in the normal weight group, but not in the overweight or obese groups [model 2: *β* 0.67 (95% CI 0.13; 1.21)]. Second, analyses were stratified based on significant interactions (*p* < 0.10). A significant interaction was observed between at least one of the macronutrients and sex (*p* value range 0.08–0.35), and no significant interaction was observed for age (*p* value range 0.12–0.37). Stratification by sex using the nutrient residual method did not alter the results (S2). Third, a sensitivity analysis excluding all nutritional components from the frailty index did not alter the direction or strength of the association of fat and protein with frailty (results not shown).

## Discussion

This study did not observe an association between total carbohydrates and total fats with frailty over time. A positive association between mono-unsaturated fatty acids intake and frailty in the total population was observed. Furthermore, an association between protein intake and more frailty over time was seen, but only among those with normal weight. This association was mainly driven by animal protein which was associated with a higher frailty index score over time. Moreover, higher protein intake at the expense of a concomitant lower intake of carbohydrates or fat was associated with more frailty over time.

Comparison of our results with published data is challenging, because data on the association between nutrition and frailty are scarce. A recent review also emphasized that most studies focused on the association between protein intake and the physical domain of frailty [12]. Far less is known for other domains of frailty: cognition, mood, social health, and comorbidity. The frailty phenotype is physically orientated, and is distinct from disabilities, chronic diseases, cognition, and mental health, whereas the frailty index does include these health domains. Moreover, other studies used different definitions of frailty or overall health.

In our study, we did not find an association between carbohydrate intake and frailty after full adjustment. To our knowledge, no studies are known for assessing the association between carbohydrates and frailty. Furthermore, no association between overall total fat intake and frailty was seen in our study. Nevertheless, we did observe an association between mono-unsaturated fatty acids intake and more frailty over time in the total population. This result was unexpected as mono-unsaturated fatty acids are generally known to be beneficial for several components of frailty including cognition [45]. However, important contributors to total mono-unsaturated fatty acids intake are meat products, added fats, and dairy products [46]. In line with our results, Hodge et al. showed in a prospective cohort study that a dietary pattern, high meat, and fatty products were associated with worsening health [47].

We did not observe an association between total protein intake and frailty in the full population. The possible beneficial effect of high protein intake on muscle function may be omitted by a possible negative association between protein and other health domains including digestive, renal, and vascular domains [48]. Also, high dietary protein intake is often associated with a low diet quality, which might have a negative effect on the frailty status [23–25, 49]. In our study, we did observe an association between high intake of protein at the expense of carbohydrates and more frailty over time. This is in line with two systematic reviews which concluded that high protein intake but a low carbohydrate intake was associated with higher all-cause mortality risk [19, 20]. Also, we did observe an association between higher protein intake and increased frailty scores among participants with a normal weight, but not in participants who were overweighed or obese. High protein diet is associated with lower food intake, lower body weight, and body adiposity [14–17]; this might explain that we did observe an association in normal weight participants, but not in overweight or obese. Persons with overweight or obesity have in general a high nutritional intake and, therefore, comply with dietary guidelines; however, the macronutrient composition might be less important for older adults suffering from overweight or obesity as an overall unhealthy diet mediates the association between the macronutrient composition and frailty. In our study, the association between protein and higher frailty status over time is mainly driven by higher intake of animal protein. A diet high in animal protein intake (such as meat) contributes to a higher dietary acid load. Because a high dietary acid load is associated with different chronic diseases, this might contribute to a higher frailty index score [50, 51]. Whereas high intake of plant protein is associated with a healthy dietary pattern which is, in turn, associated with a lower frailty status [24, 25, 27, 52].

This study has numerous strengths. To the best of our knowledge, this is the first study investigating the longitudinal association between macronutrient intake and the frailty index. Additionally, the comprehensive data collection allowed us to control for many confounders. Furthermore, the large sample size and multiple imputation procedure contributed to a more precise estimate of the association. Most studies on protein did not take into account the role of energy intake and other macronutrients in the diet and it is, therefore, unclear whether the onset and progression of frailty is affected by higher absolute or relative intake of protein, and for relative measure, if this is explained by lower intake of carbohydrates or fat. By taking total energy intake into account, the interpretation of the role of specific macronutrients will improve [40]. The present study used different statistical methods to take the possible modifying and confounding effect of total energy into account, giving us more insight into the association between macronutrient intakes and frailty.

Despite these strengths, there are several limitations to consider. First, since there is no consensus on the definition of frailty, there are a variety of instruments to assess frailty and overall health which limits the comparability of our results. Measures of frailty show important differences with the frailty index, making a direct comparison with previous literature complex. Second, participants had relatively low frailty indices, and in many participants (37%), the frailty index became lower over time, whereas it was expected to increase. This might be explained, because a relatively healthy population participated in this study, which might have been expected as older adults who are frail or more vulnerable are less likely to participate in the study [53–55]. This may have led to less strong associations. This limits the generalizability of our study results in more vulnerable populations. Third, because this study included multiple waves of the Rotterdam Study, different FFQs were used to measure dietary intake. Nevertheless, the use of an up-to-date FFQ to assess dietary intake has been advised to take into account the availability of new foods and new food composition [56]. Finally, results may have been influenced by report bias as persons may give more socially desirable answers and exaggerate the consumption of healthy foods which might increase our estimate of the effect [57].

In conclusion, our study contributed to the knowledge on the association between macronutrients and frailty over time. The intake of fat and carbohydrates did not contribute to the association between the macronutrient composition of the diet and overall health, measured by the frailty index. High protein intake, specifically animal protein intake, is associated with more frailty in a relatively healthy older adult population. Further research is needed on the association between protein intake and multidimensional frailty, focused on the source of protein, and on more vulnerable populations.

## Electronic supplementary material

Below is the link to the electronic supplementary material.

Supplementary material 1 (DOCX 26 kb)
