# Supplementary material for: Macronutrient intake and frailty: the Rotterdam Study
Source: Eur J Nutr. 2019 Nov 14;59(7):2919–28. doi: 10.1007/s00394-019-02131-0 (PMC7501120; doi:10.1007/s00394-019-02131-0)
Supplement: Supplementary file 1 — Supplementary material 1 (DOCX 26 kb) [file 394_2019_2131_MOESM1_ESM.docx]

**Macronutrient intake and frailty: the Rotterdam Study**

European journal of Nutrition

Eline Verspoor1,2, Trudy Voortman1, Frank J.A. van Rooij1, Fernando Rivadeneira1,3, Oscar H. Franco1,5, Jessica C. Kiefte-de Jong1,4, Josje D. Schoufour1,3,6

1 Department of Epidemiology, Erasmus University Medical Center, Postbus 2040, 3000CA, Rotterdam, the Netherlands

2 Department of Public Health, Erasmus University Medical Center, Postbus 2040, 3000CA, Rotterdam, the Netherlands

3 Department of Internal Medicine, Erasmus University Medical Center, Postbus 2040, 3000CA, Rotterdam, the Netherlands

4 Department of Public Health and Primary Care, Leiden University Medical Center/LUMC Campus, The Hague, The Netherlands

5 Institute of Social and Preventive Medicine (ISPM), University of Bern, Bern, Switzerland

6 Faculty of Sports and Nutrition, ACHIEVE - Centre of Applied Research, Faculty of Health, Amsterdam University of Applied Sciences, Amsterdam, the Netherlands

**Corresponding author**

Trudy Voortman, Erasmus MC, Department of Epidemiology, P.O. Box 2040, 3000CA, Rotterdam, the Netherlands, Phone +31 (0)10 70 43536, e-mail: [trudy.voortman@erasmusmc.nl](mailto:trudy.voortman@erasmusmc.nl)

**S1.** Longitudinal association between macronutrient intake and the frailty index using energy decomposition method in a Dutch middle-aged and older population

| Macronutrient | Population | | Model 1 | |  | Model 2 | |  | Model 3 | |
| --- | --- | --- | --- | --- | --- | --- | --- | --- | --- | --- |
|  |  | | β | (95% CI) |  | β | (95% CI) |  | β | (95% CI) |
| Carbohydrate (per 100 kcal) | | Total population | **-0.08*** | **-0.15; -0.01** |  | -0.06 | -0.14; 0.02 |  | -0.03 | -0.11; 0.05 |
|  | | Normal weight | -0.13 | -0.26; 0.004 |  | -0.09 | -0.23; 0.06 |  | -0.08 | -0.23; 0.06 |
|  | | Overweight | -0.05 | -0.16; 0.06 |  | 0.004 | -0.12; 0.13 |  | 0.02 | -0.11; 0.14 |
|  | | Obesity | -0.03 | -0.18; 0.11 |  | -0.09 | -0.25; 0.07 |  | -0.07 | -0.23; 0.09 |
| Fat (per 100 kcal) | | Total population | **0.12*** | **0.03; 0.20** |  | 0.09 | -0.01; 0.18 |  | 0.08 | -0.01; 0.17 |
|  | | Normal weight | 0.12 | -0.03 0.28 |  | 0.05 | -0.13; 0.22 |  | 0.05 | -0.13; 0.22 |
|  | | Overweight | **0.14*** | **0.01; 0.26** |  | 0.11 | -0.04; 0.25 |  | 0.10 | -0.04; 0.24 |
|  | | Obesity | 0.08 | -0.08; 0.24 |  | 0.12 | -0.06; 0.29 |  | 0.11 | -0.07; 0.28 |
| Protein (per 100 kcal) | | Total population | 0.01 | -0.27; 0.29 |  | 0.16 | -0.13; 0.44 |  | 0.03 | -0.26; 0.32 |
|  | | Normal weight | 0.48 | -0.06; 1.01 |  | **0.67*** | **0.13; 1.21** |  | **0.66*** | **0.12; 1.20** |
|  | | Overweight | -0.25 | -0.68; 0.18 |  | -0.12 | -0.55; 0.31 |  | -0.16 | -0.59; 0.28 |
|  | | Obesity | -0.31 | -0.88; 0.25 |  | -0.23 | -0.82; 0.35 |  | -0.35 | -0.94; 0.23 |

^Values represent the difference in frailty index score per every increase of 100 kcal macronutrient intake, while intake of all other macronutrients is held constant, with their corresponding 95% Confidence Intervals (CI). Model 1 (basic model) was adjusted for age (continuous), sex (categorical), length of follow-up (continuous), frailty index at baseline (continuous), cohort (categorical), and kcal (continuous). Model 2 (confounder model) was additionally adjusted for education (categorical), physical activity (continuous), income (categorical), living situation (categorical), occupational situation (categorical), fiber intake (continuous), and alcohol intake (categorical). Model 3 (intermediate model) was additionally adjusted for BMI (continuous). *Statistically significant at a p-value <0.05.^

**S2.** Longitudinal association between macronutrient intake and the frailty index using nutrient residual method in a Dutch middle-aged and older population by sex.

| Macronutrient | Population | Model 2 | |
| --- | --- | --- | --- |
|  |  | β | (95% CI) |
| Carbohydrates (per 10 g/d) | Total population | -0.05 | -0.10; 0.003 |
|  | Women | -0.07 | -0.15; 0.003 |
|  | Men | -0.04 | -0.11; 0.04 |
| Fat (per 10 g/d) | Total population | 0.11 | -0.01; 0.23 |
|  | Women | 0.16 | -0.003; 0.33 |
|  | Men | 0.06 | -0.12; 0.25 |
| Protein (per 10 g/d) | Total population | 0.07 | -0.06; 0.20 |
|  | Women | 0.08 | -0.10; 0.26 |
|  | Men | 0.08 | -0.10; 0.27 |

^Values represent the difference in frailty index score per every increase of 10 gram macronutrient intake, keeping the energy intake constant (iso-energetic) with their corresponding 95% Confidence Intervals (CI). All models were adjusted for age (continuous), sex (categorical), length of follow-up (continuous), frailty index at baseline (continuous), cohort (categorical), kcal (continuous), education (categorical), physical activity (continuous), income (categorical), living situation (categorical), occupational situation (categorical), fiber intake (continuous), and alcohol intake (categorical). *Statistically significant at a p-value <0.05.^
